# The complete mitochondrial genome and phylogenetic position of *Triplophysa qiubeiensis* (Cypriniformes: Nemacheilidae)

**DOI:** 10.1080/23802359.2026.2657110

**Published:** 2026-04-15

**Authors:** Yizhu Chen, Yuansheng Zhu, Yue Wang, Dengqiang Wang

**Affiliations:** ^a^ Scientific Institute of Pearl River Water Resources Protection, Guangzhou, China; ^b^ Hongshui River Rare Fish Conservation Center, Guiping, China; ^c^ Yangtze River Fisheries Research Institute, Chinese Academy of Fishery Sciences, Wuhan, China

**Keywords:** Nemacheilidae, cave-dwelling fish, mitochondrial DNA, phylogenetic analysis

## Abstract

In this study, the complete mitochondrial genome of *Triplophysa qiubeiensis* (Cypriniformes: Nemacheilidae) was determined using the Illumina Novaseq 6000 platform. The mitogenome is 16,584 bp in length, and exhibits a gene composition and arrangement typical of the teleost fishes, containing 13 protein-coding genes, 22 tRNA genes, two ribosomal RNA genes, and a control region. The overall nucleotide composition was 30.63% A, 27.18% T, 16.37% G, and 25.82% C. Phylogenetic analysis based on complete mitogenome sequences placed *T. qiubeiensis* within a clade of *Triplophysa* species primarily distributed in the Yunnan-Guizhou Plateau of southwestern China.

## Introduction

*Triplophysa* fishes, belonging to family Nemacheilidae, order Cypriniformes, are widely distributed across the Tibetan Plateau and its surrounding areas. Their speciation and evolutionary history are thought to be closely related to the uplift of the Tibetan Plateau (Zhu 1989; Wu and Wu 1992). As of 2024, FishBase has recorded 166 *Triplophysa* species (Froese and Pauly, 2024). These species inhabit diverse habitats such as rivers, lakes and caves at different altitudes, reflecting remarkable ecological and taxonomic diversity. However, studies on individual molecular markers of *Triplophysa* fishes have all exhibited relatively low discriminatory ability (Wang et al. 2020; Zhou et al. 2025). To date, the complete mitochondrial genomes of dozens of *Triplophysa* species have been published. (Wang et al. 2016; Yang et al. 2020; Wang et al. 2021; Peng et al. 2023). It is hoped that these more comprehensive data can clarify the interspecific relationships within the *Triplophysa* fishes.

*T. qiubeiensis* Li & Yang, 2008 was first discovered in a cave of Qiubei County, Yunnan province, China during field surveys in 2002 and 2006, and formally described in 2008 (Li et al. 2008). This cave-dwelling fish is characterized by highly reduced eyes, a dorsal fin with seven spiny rays, a forked tail fin, and a scaleless body. In this study, we reported the complete mitochondrial genome of *T. qiubeiensis* and reconstructed its phylogenetic position within the genus *Triplophysa*.

## Materials and methods

**Figure 1.**
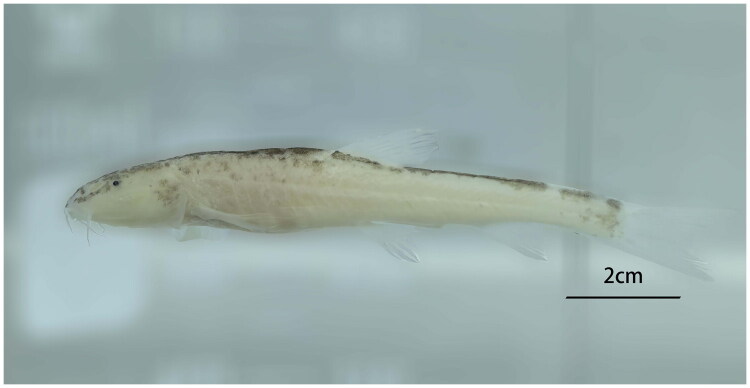
Photograph of *Triplophysa qiubeiensis* taken by Yizhu Chen. Specimen was collected in a cave from Shuanglongying county, Yunnan province, China on 10 July 2024 and deposited at Hongshui River Rare Fish Conservation Center under voucher number GYQ2024071005. Eyes highly degraded, the caudal fin is forked and number of branched dorsal–fin ray is 7.

We collected the five specimens of *T. qiubeiensis* from a cave in Qiubei county, Yunnan province, China (24°20′N, 104°11′E) on 10 July 2024 (Figure 1). We identified the species according to the characteristics in the key table published by Li et al. and found that the species’ eyes degenerated, a pinhole remained in the blinded eye, a cone-shaped head, and a triangular nasal flap, which were consistent with the characteristics of *T. qiubeiensis* (Li et al. 2008). The specimens were deposited at Hongshui River Rare Fish Conservation Center (Yizhu Chen, 18738573160@163.com) under voucher number GYQ2024071001∼GYQ2024071005.

Total genomic DNA was extracted from muscle tissue using a tissue genomic DNA extraction kit (Tiangen, Beijing, China) and a DNA sequencing library with an insert fragment of 350 bp was constructed using the Nextera XT DNA library preparation kit (Illumina, San Diego, CA), followed by dual-end sequencing using Illumina Novaseq 6000 platform. Raw data was filtered using fastp (Chen 2023) with the following criteria: removing reads with >5% N bases, reads containing ≥50% low-quality bases (quality value ≤5), and reads with adapter contamination. This yielded high-quality ‘clean data.’ The clean data was *de novo* assembled using SPAdes v.3.14.1(Prjibelski et al. 2020) into mitochondrial contigs. Then, we compared the assembly outcomes with the reference genome of *T. jianchuanensis* (GenBank accession number: OQ603602) to determine whether the assembly formed a circular structure, as well as the orientation of the contigs, and the position of the start base. The assembled complete mitochondrial genome was annotated using the MITOS (Bernt et al. 2013) software. Average sequencing depth was 1471.26×(Supplementary Figure S1), as calculated using SAMtools (Li et al. 2009). A circular map of the mitochondrial genome was generated using Proksee online (Grant et al. 2023).

For phylogenetic analysis, we used 47 mitochondrial genomes, including 43 *Triplophysa* sequences to determine the phylogenetic position of *T. qiubeiensis*. Species from the genera *Barbatula* and *Homatula* were used as the outgroup. We aligned all sequences using Clustal W in MEGA 7.0 (Kumar et al., 2016) and reconstructed phylogenetic relationships using the IQ-TREE web server (Trifinopoulos et al. 2016), with 2000 ultrafast bootstrap replicates. The best-fit substitution model GTR+ I + G was selected using ModelFinder in IQ-TREE based on the Bayesian information criterion (BIC).

## Results

### Mitogenome organization

**Figure 2.**
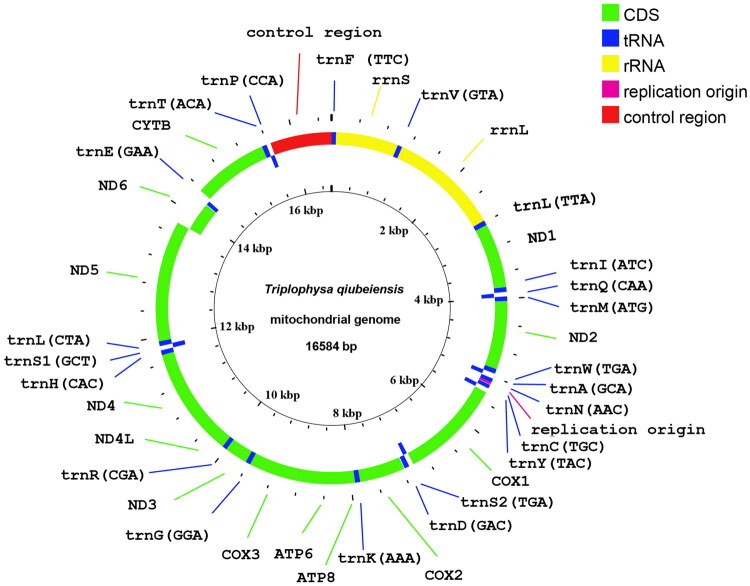
Complete mitochondrial genome organization and gene arrangement of *Triplophysa qiubeiensis*. Genes coded on the H strand are directed to the outer ring, and the genes coded on the L strand are indicated in the interior of the ring.

The complete mitochondrial genome of *T. qiubeiensis* (GenBank accession number: PQ563459) is 16,584 bp in length. The overall nucleotide composition is 30.63% A, 27.18% T, 16.37% G, and 25.82% C. The genome contains 13 protein-coding genes, 22 tRNA genes, two rRNA genes, and a control region (D-loop) (Figure 2). Of these, 28 genes are encoded on the Heavy-Strand (H-strand), whereas the *ND6* gene and eight tRNA genes (*trnQ*, *trnA*, *trnN*, *trnC*, *trnY*, *trnS2*, *trnE*, and *trnP*) are encoded on the Light-Strand (L-strand).

Like most vertebrates, 12 of the 13 protein-coding genes initiate with the ATG codon, while *COX1* uses GTG as its start codon. Seven protein-coding genes (*ND1, COX1, ATP8*, *ATP6*, *ND4L ND5* and *ND6*) terminate with the complete TAA stop codon, and the remaining six genes (*ND2*, *COX2*, *COX3, ND3*, *ND4*, and *CYTB*) use incomplete stop codons (T or TA). The 22 tRNA genes range in length from 66 to 76 bp. There are six gene overlapping regions ranging from 1 to 10 bp, and twelve intergenic spacer regions ranging from 1 to 31 bp (Figure 2).

### Phylogenetic analysis

**Figure 3.**
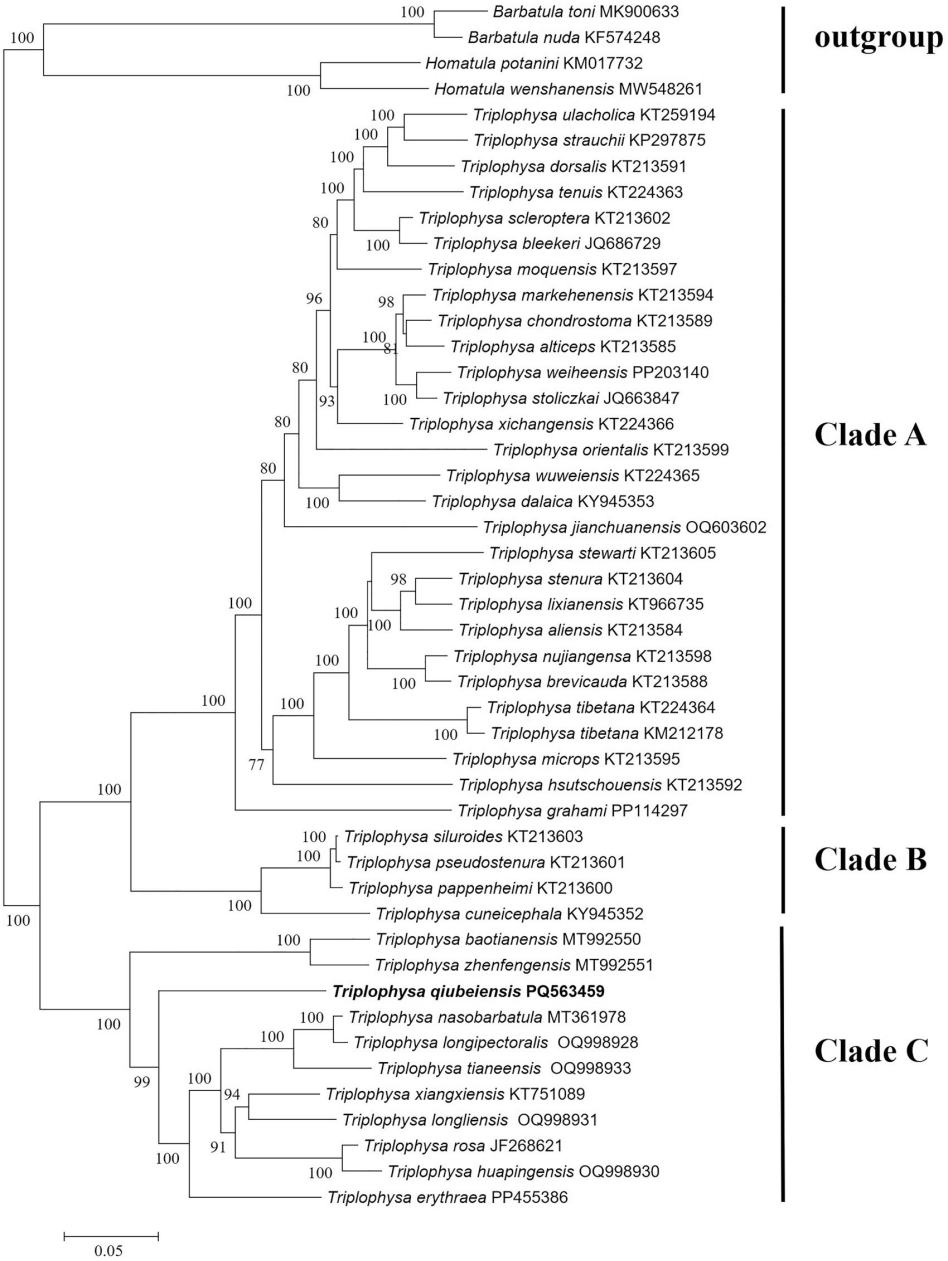
Maximum Likelihood (ML)phylogenetic tree based on complete mitochondrial genome of 43 *Triplophysa* species. *Barbatula toni* MK900633 (Yang et al. 2019), *B. nuda* KF574248 (Zhao et al. 2015), *Homatula potanini* KM017732 (Que et al. 2016) and *H. wenshanensis* MW548261 (Cui et al. unpublished) are used as an outgroup. GenBank accession numbers and bootstrap values of nodes are shown on the tree. The boldface indicates new sequence generated in this study. The *Triplophysa* sequences used are as follows: *T. ulacholica* KT259194 (Wang et al. 2016), *T. strauchii* KP297875 (Kanu et al. 2016), *T. dorsalis* KT213591 (Wang et al. 2016), *T. tenuis* KT224363 (Wang et al. 2016), *T. scleroptera* KT213602 (Wang et al. 2016), *T. bleekeri* JQ686729 (Si et al. unpublished), *T. moquensis* KT213597 (Wang et al. 2016), *T. markehenensis* KT213594 (Wang et al. 2016), *T. chondrostoma* KT213589 (Wang et al. 2016), *T. alticeps* KT213585 (Wang et al. 2016), *T. weiheensis* PP203140 (Niu et al. unpublished), *T. stoliczkai* JQ663847 (Li et al. 2013), *T. xichangensis* KT224366 (Wang et al. 2016), *T. orientalis* KT213599 (Wang et al. 2016), *T. wuweiensis* KT224365 (Wang et al. 2016), *T. dalaica* KY945353 (Feng et al. unpublished), *T. jianchuanensis* OQ603602 (Peng et al. unpublished), *T. stewarti* KT213605 (Wang et al. 2016), *T. stenura* KT213604 (Wang et al. 2016), *T. lixianensis* KT966735 (Wang et al. 2016), *T. aliensis* KT213584 (Wang et al. 2016), *T. nujiangensa* KT213598 (Wang et al. 2016), *T. brevicauda* KT213588 (Wang et al. 2016), *T. tibetana* KT224364 (Wang et al. 2016), *T. tibetana* KM212178 (Ma and Yang, unpublished), *T. microps* KT213595 (Wang et al. 2016), *T. hsutschouensis* KT213592 (Wang et al. 2016), *T. grahami* PP114297 (Xu et al. 2024), *T. siluroides* KT213603 (Wang et al. 2016), *T. pseudostenura* KT213601 (Wang et al. 2016), *T. pappenheimi* KT213600 (Wang et al. 2016), *T. cuneicephala* KY945352 (Feng et al. unpublished), *T. baotianensis* MT992550 (Wang et al. 2021), *T. zhenfengensis* MT992551(Carraretto et al. 2020), *T. nasobarbatula* MT361978 (Luo et al. unpublished), *T. longipectoralis* OQ998928 (Zhang et al. unpublished), *T. tianeensis* OQ998933 (Zhang et al. unpublished), *T. xiangxiensis* KT751089 (Wang et al. 2017), *T. longliensis* OQ998931 (Zhang et al. unpublished), *T. rosa* JF268621 (Wang et al. 2012), *T. huapingensis* OQ998930 (Zhang et al. unpublished), *T. erythraea* PP455386 (Wang and Dong, unpublished).

The ML phylogenetic trees constructed from the complete mitochondrial genome data showed that all *Triplophysa* species clustered into three major clades with 100% bootstrap support (Figure 3). *T. qiubeiensis* was nested within clade C, which includes *T. baotianensis*, *T. zhenfengensis*, *T. qiubeiensis*, *T. erythraea*, *T. nasobarbatula*, *T. longipectoralis*, *T. tianeensis*, *T. xiangxiensis, T. longliensis*, *T. rosa, T. huapingensis*. Most species in this clade are cave-dwelling and are predominantly distributed on the Yunnan-Guizhou Plateau in southwestern China.

## Discussion and conclusion

Cave fish are aquatic species that complete their entire life cycle in enclosed cave environment. Adaptation to cave habitats has driven extensive morphological modifications in cave fish, such as eye degeneration, loss of body pigmentation, and advanced olfactory capabilities (Hinaux et al. 2016; Blin et al. 2018; Borowsky 2018). Owing to their wide distribution and strong adaptability capacity, cave-dwelling *Triplophysa* species have become an ideal model for studying the adaptive evolution of cave fish (Song et al. 2024). Qiubei County is characterized by a typical karst landform, which is accompanied by extensive karst caves, numerous underground rivers, and a complex hydrological system. Geographically, the county is situated in a high-altitude region, with an average elevation of more than 1,000 meters. The cave water in this area is characterized by low temperature and low dissolved oxygen content, which leads to a relatively scarce aquatic biota. Up to now, *T. qiubeiensis* has been exclusively recorded in the karst caves within Qiubei County, showing a narrow distribution range and a small population size (Li et al. 2009). The *T. qiubeiensis*, as described in this study, exhibits typical cave-adaptive traits, including eyes degeneration and reduced body pigmentation.

We report for the first time the complete mitogenome of *T. qiubeiensis*, comprising 16,584 bp in length. Its gene arrangement, structure and content are consistent with those of other teleost fish (Saitoh et al. 2006; Wang et al. 2016; Yang et al. 2020; Wang et al. 2021; Peng et al. 2023). Our phylogenetic analysis confirms the monophyly of the genus *Triplophysa*, which aligns with the findings of previous studies (Wang et al. 2016, 2021, 2024; Song et al. 2025). Within *Triplophysa*, *T. qiubeiensis* is closely related to a group of cave-dwelling congeners that inhabit the Yunnan-Guizhou Plateau in southwestern China. In conclusion, our findings provide valuable molecular data for future research on the evolutionary history, genetic diversity and population genetics of cave-dwelling *Triplophysa* species, including *T. qiubeiensis*.

## Supplementary Material

Supplementary.docx

## Data Availability

The genome sequence data that support the findings of this study are openly available in GenBank of NCBI at https://www.ncbi.nlm.nih.gov/ under the accession number PQ563459. The associated BioProject, Bio-Sample, and SRA numbers are PRJNA1184832, SAMN44677753, and SRR31311005, respectively.
