# Supplementary material for: The complete mitochondrial genome and phylogenetic position of Triplophysa qiubeiensis (Cypriniformes: Nemacheilidae)
Source: Mitochondrial DNA B Resour. 2026 Apr 15;11(5):614–8. doi: 10.1080/23802359.2026.2657110 (PMC13084831; doi:10.1080/23802359.2026.2657110)
Supplement: Supplementary.docx [file TMDN_A_2657110_SM5460.docx]

**Supplemental Material**

**The complete mitochondrial genome and phylogenetic position of *Triplophysa qiubeiensis* (Cypriniformes: Nemacheilidae)**

Yizhu Chen^a, b^, Yuansheng Zhu^a, b^, Yue Wang^a, b^, Dengqiang Wang^c, *^

a. Scientific Institute of Pearl River Water Resources Protection, Guangzhou 510611, China

b. Hongshui River Rare Fish Conservation Center, Guiping 537200, China

c. Yangtze River Fisheries Research Institute, Chinese Academy of Fishery Sciences, Wuhan 430223, China

* Corresponding author: wdq@yfi.ac.cn


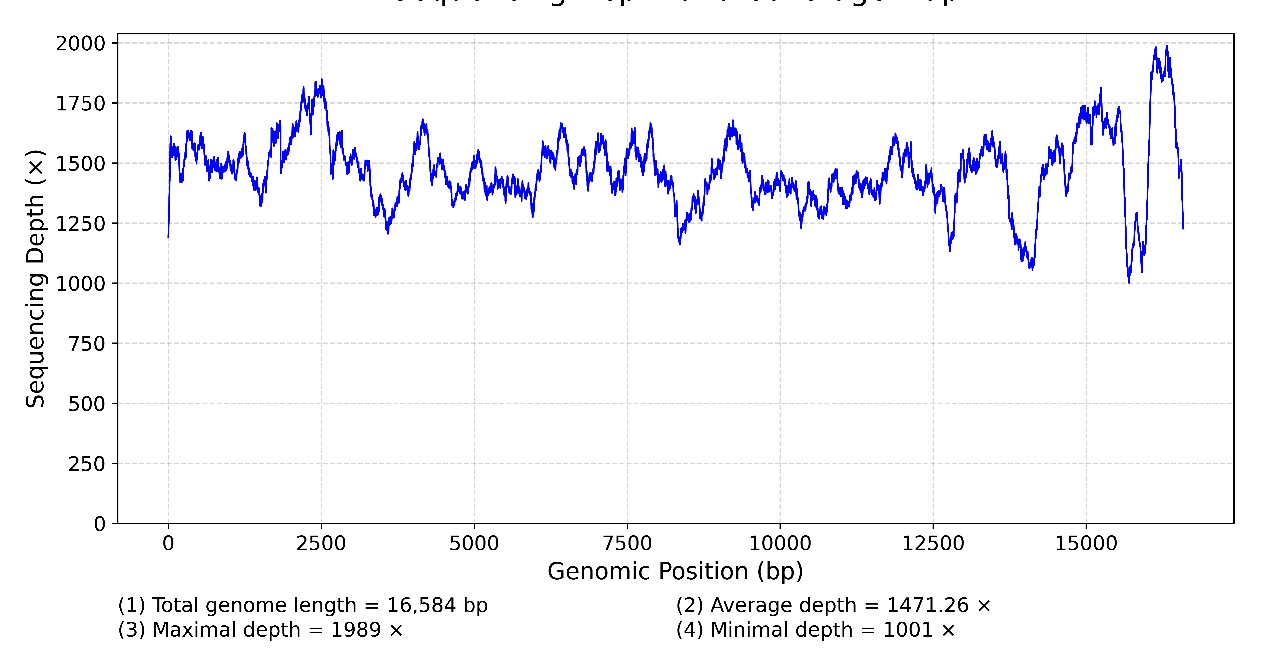


**Figure S1.** Sequencing depth and coverage map of *Triplophysa qiubeiensis* mitochondrial genome. The results shows the average sequencing depth is 1,471.26× and the genome size is 16, 584 bp.
